# Rehabilitating the sick role: post-surgical experiences of high risk women who undergo risk reducing mastectomy

**DOI:** 10.1186/1897-4287-10-S2-A6

**Published:** 2012-04-12

**Authors:** N Hallowell, L Heiniger, B Baylock, M Price, P Butow, kConFab Psychosocial Group

**Affiliations:** 1 Institute of Health & Society, Newcastle University, UK; 2 Centre for Medical Psychology and Evidence-based Decision-making (CeMPED), University of Sydney, Australia

## 

Much has been written about the impact of risk-reducing breast and ovarian surgery on quality of life and high-risk women’s surgical decision-making, but much less is known about how this group experiences these elective procedures. In this paper we describe the ways in which women who have undergone risk-reducing breast surgery (+/- ovarian surgery) describe their surgical experiences. Data was collected during in-depth interviews with 21 Australian women from the kConFab Psychosocial study who had undergone risk-reducing mastectomy in the previous three years. Interview questions centred on decision-making, information needs, perceived costs and benefits of surgery, risk perception, pre-surgery expectations and knowledge, the surgery experience and convalescence, and overall satisfaction with surgical decision. When describing their experiences of surgery and convalescence women drew on two main narratives in which they described the immediate impact of surgery on convalescence and either embraced or vigorously rejected the sick role (Parsons, 1951). The extent to which women appeared to accept/reject the sick role appeared to be related to the amount of support/lack of support they received from families, friends and healthcare professionals. We conclude by arguing that the concept of the sick role can provide us with some insight into high-risk women’s’ experience of surgery and convalescence.

